# Endoscopic ultrasound (EUS) guided choledocoduodenostomy in a patient with irresecable pancreas cancer and biliar obstruction using a luminal apposing metal stent (LAMS)

**DOI:** 10.1016/j.ijscr.2020.07.041

**Published:** 2020-07-24

**Authors:** Renzo Pinto-Carta, Jaime Solano, Luis Felipe Cabrera, Alvaro Sanchez, Luisa Moreno, Mauricio Pedraza

**Affiliations:** a Department of Gastroenterology and Digestive Endoscopy, Santa Fe Foundation University Hospital of Bogotá, Bogotá, Colombia; b Department of Surgery, Jose Felix Patiño, Fundación Santa Fe de Bogotá, Bogotá, Colombia; c Department of General Surgery, University El Bosque, Bogotá, Colombia; d Department of Internal Medicine, University El Bosque, Bogotá, Colombia; e Department of Medicine, Universidad Los Andes, Bogotá, Colombia

**Keywords:** Endoscopic ultrasound, Endoscopic ultrasound-guided biliary drainage, Choledocoduodenostomy, Luminal apposing stent (LAMS), Biliary obstruction, Pancreatic cancer

## Abstract

The endoscopic retrograde cholangiopancreatography (ERCP) is the election treatment of biliary obstruction. EUS-guided biliary drainage (EUS-BD) has emerged as an alternative to PTBD with a high technical and clinical success rate, low risk of complications and a better quality of life for the patient. Case presentation we present the first case in Colombia of EUS-guided biliary drainage using luminal apposing metal stent (LAMS) in a patient with unresectable pancreatic adenocarcinoma with biliary obstruction in who ERCP was failed due to neoplastic invasion of the Vater papilla. Discussion Transhepatic biliary drainage has been the most common procedure for treatment of malignant biliary obstruction in cases which ERCP fails due to tumor infiltration of the duodenum or the Vater papilla. During the last decade the development of endoscopic ultrasound (EUS) has implied an alternative for biliary drainage in cases of failed ERCP, demonstrating advantages over Trans hepatic biliary drainage Conclusion Considering that EUS-CD is a safe procedure, with a high rate of technical and clinical success, low risk of complications.

## Highlights

• The ERCP is the election treatment of biliary obstruction syndrome.
• EUS-DB is an alternative to PTBD with high technical and clinical success rate.
• EUS-DB is a safe and feasible procedure, providing a better quality of life to the patient.

## 1 Introduction

The endoscopic retrograde cholangiopancreatography (ERCP) is the election treatment of biliary obstruction secundary to benign or malignant etiology, but this can fail in 5–10% of cases due to anatomical alterations of the Vater papilla, intradiverticular papilla, neoplastic invasion of the duodenum or altered anatomy. Traditionally in these cases, the second choice treatment was transhepatic biliary drainage (PTBD), with a high complication rate of up to 33%, including bleeding, infections, catheter displacement, bile leakage and which significantly alters the quality of life of the patient.

EUS-guided biliary drainage (EUS-BD) has emerged as an alternative to PTBD with a high technical and clinical success rate, low risk of complications and a better quality of life for the patient. There are two techniques, choledocoduodenostomy (EUS-CDS) and hepaticogastrostomy (EUS-HGS).

EUS-BD was first described by Giovannini et al. in 2001 [1], since then, many studies have been published demonstrating high rates of technical and clinical success (95% and 97% respectively) and low risk of complications.

The development of dedicates devices such as luminal apposing metal stent (LAMS) and others such as GIOBOR, has displaced the use of self-expanding metal stents (SEMS) with which the risks of complications such as bile leakage, stent displacement, pneumoperitoneum were increased, allowing to perform this procedure with greater security and minimizing the risks. This work has been reported in line with the SCARE criteria [12].

## 2 Methodology

We report the first case in Colombia of EUS-CDS using LAMS of 8 mm × 8 mm HOT AXIOS stent (Boston Scientific Corp, Marlborough, Mass, USA) in a patient with unresectable pancreatic adenocarcinoma with biliary obstruction in who ERCP was failed due to neoplastic invasion of the Vater papilla.

## 3 Case report

**Fig. 1:**
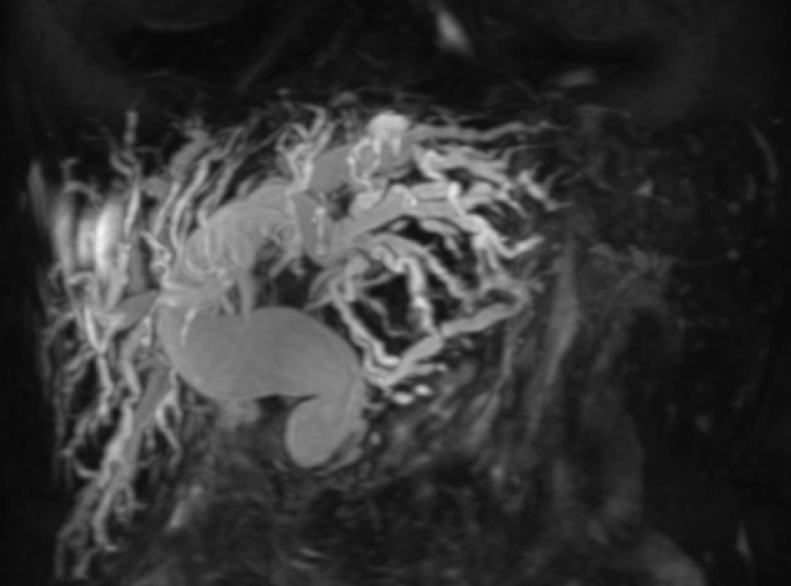
MRCP image shows intra and extrahepatic biliary dilation secondary to tumor lesion of the head of the pancreas.

**Fig. 2:**
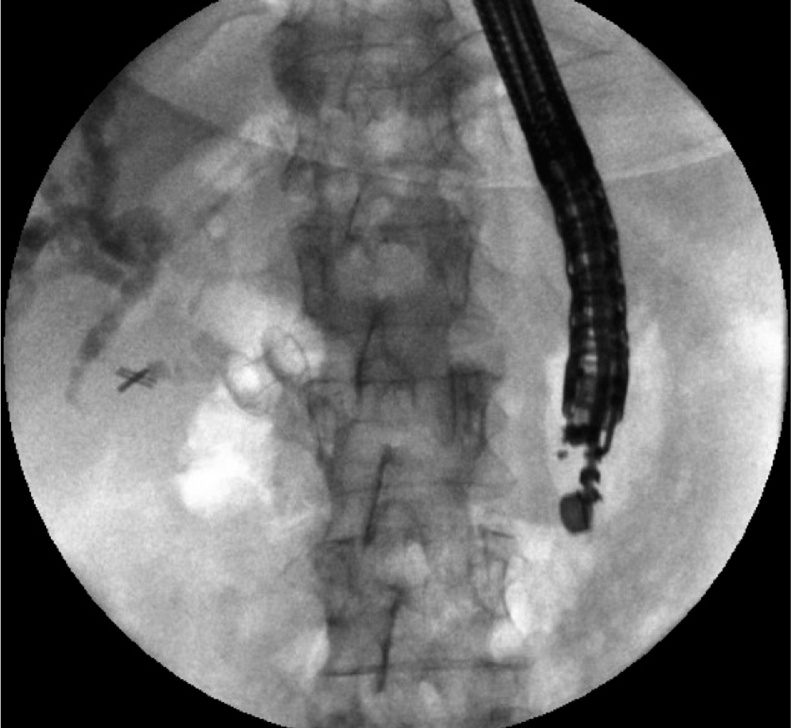
Fluoroscopic image observing LAMS between duodenal bulb and bile duct.

**Fig. 3:**
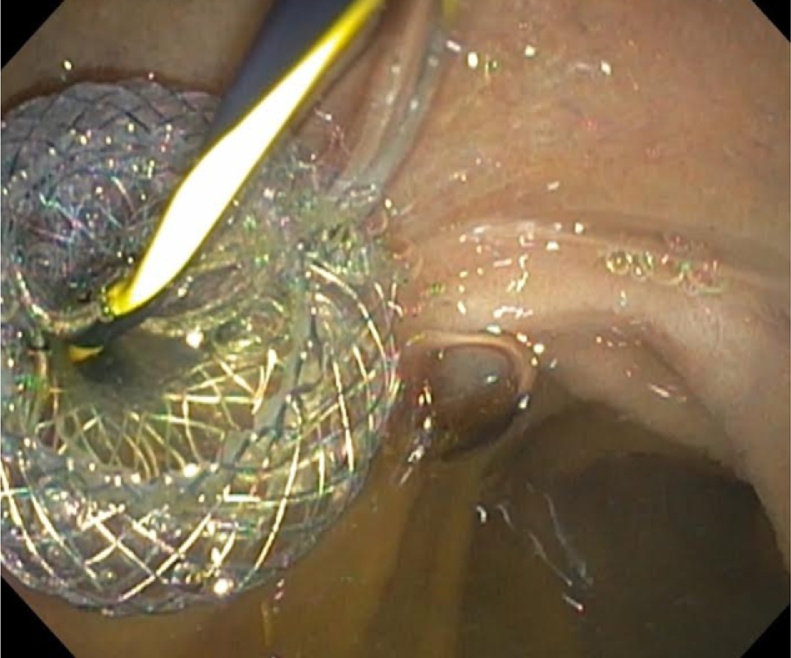
Endoscopic vision shows LAMS (HOT AXIOS) in duodenal bulb with adequate biliary drainage.

**Fig. 4:**
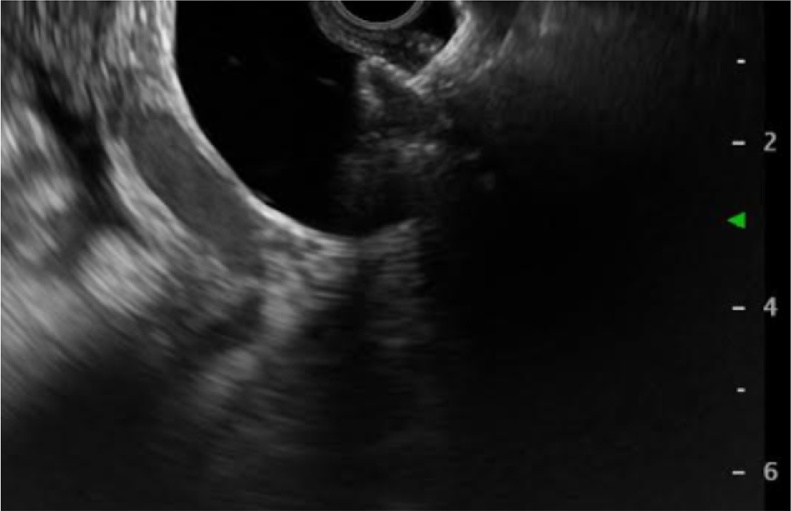
Endoscopic ultrasound vision shows the distal flange of LAMS (HOT AXIOS) in to the CBD.

66-year-old female patient with unresectable pancreatic head adenocarcinoma who consults to emergency department for presenting progressive jaundice without abdominal pain. Laboratories showed total bilirubin (BT) at 18 mg/dl, hemogram without leukocytosis. MRCP showed common bile duct (CBD) obstruction secondary to pancreatic head tumor with CBD and the intrahepatic bile duct dilatation Fig. 1. ERCP was performed without access to the bile duct due to tumor infiltration of the second duodenal portion and the Vater papilla. Therefore, we decided to perform an EUS-CD using a LAMS (HOT AXIOS) to biliary drainage. Fluoroscopic image observing LAMS between duodenal bulb and bile duct (Fig. 2), endoscopic vision shows LAMS (HOT AXIOS) in duodenal bulb with adequate biliary drainage (Fig. 3). Endoscopic ultrasound vision shows the distal flange of LAMS (HOT AXIOS) in to the CBD (Fig. 4).

The procedure was performed after obtaining informed explaining the risks, benefits and alternatives.

**Fig. 5:**
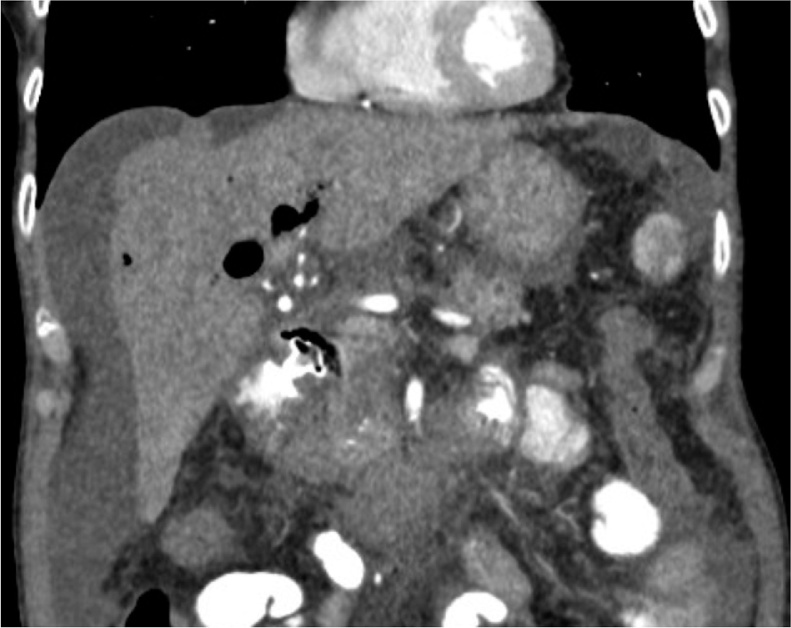
CT scan coronal view shows LAMS in proper position.

The patient was followed daily for 24 h intrahospitally, abdominal CT scan was performed where the adequate position of the LAMS and the presence of complications were verified, (Fig. 5) she was discharged and 30 days later she was evaluated observing a decrease of more than 50% of the BT compared to the previous one to the procedure, there were no complications).

## 4 EUS-CDS technique

The procedure was performed by an interventional gastroenterologist, an endoscopic ultrasound specialist with more than 5 years of experience in this technique and in the use of LAMS. It was performed under general anesthesia, with the help of fluoroscopy, positioning the patient in a left lateral position using the therapeutic linear echoendoscope Olympus GF-UCT180 connected to the EU-ME2 processor, transduodenally using LAMS (HOT AXIOS) 8 mm in diameter (Boston Scientific Corp, Marlborough, Mass, USA). Before the procedure, the International Normalized Ratio (INR) and platelet count were verified, which were in appropriate range (<1.5 and >50,000 respectively).

In the duodenal bulb the CBD was identified endosonographically, the absence of interposition of vessels using color Doppler was confirmed. The diameter of the CBD was determined which measured 30 mm and the distance between the duodenal wall and the CBD which was 5 mm. The LAMS catheter was passed through the echoendoscope working channel. An electrosurgical unit was used in 100 W pure cut mode and autocut mode, effect 5 (ERBE Electrosurgery, Tübingen, Germany). The catheter was introduced into the CBD under endosonographic guidance, once inside the CBD, the distal flange was deployed under the endosonographic vision, then the catheter was slightly removed until the apposition of the wall was achieved and then the proximal flange was deployed in the duodenal lumen with intracanal release, and then verifying the position by endoscopy. Subsequently, cholangiography is performed with a conical tip catheter through the LAMS, opacifying the intra and extrahepatic bile duct, dilating the entire biliary tree with adequate drainage of the contrast through the LAMS to the duodenum.

## 5 Discussion

Malignant biliary obstruction implies a poor prognosis in the short term because most patients are diagnosed at an advanced time of the disease, which limits treatment with curative intent and initiates a palliative approach process on the eve of improving quality of patients' life since the signs and symptoms they suffer such as jaundice, pruritus and intestinal obstruction can significantly deteriorate the general condition [6,9].

ERCP is the election treatment of malignant biliary obstruction which has a percentage of technical failure in those cases in which the neoplastic invasion of the papilla does not allow cannulation and therefore biliary drainage, which leads to the need to proceed with procedures such PTBD that infers a higher percentage of morbidity [1,2,9].

PTBD has been the most common procedure for treatment of malignant biliary obstruction in cases which ERCP fails due to tumor infiltration of the duodenum or the Vater papilla [1]. Even so, PTBD implies a percentage of comorbidity to be taken into account, given the need for the incessant care of the reservoir bag in need of constant washing and replacement in addition to the discomfort of permanently carrying it, which infers risk of involuntary withdrawal. It leads to sepsis, bleeding and therefore infection of the insertion site, which ultimately alters the quality of life in an attempt to improve [1].

During the last decade the development of endoscopic ultrasound (EUS) has implied an alternative for biliary drainage in cases of failed ERCP, demonstrating advantages over PTBD, such as the possibility of performing in the same intervention when the derivation is not achieved by ERCP and also less pain and infection [2,8].

The clinical success of endoscopic ultrasound guided biliary drainage with choledocoduodenostomy has been exposed in multiple series with the use of different stents and devices, to the point of being proposed as the first-line option [2]; initially it was developed the USE-CD with plastic stents getting a clinical success demonstrated in several studies such as Hara et al. who developed 94% and 100% technical and clinical success respectively, but with a percentage of stent occlusion at 163 days of 66.7% [6]. On the other hand, the risk of biliary leakage and cholangitis implies a significant percentage of morbidity which led to the use of metal stents for the use of USE-CD [6], so that studies such as Gupta and collaborators that compare the incidence of cholangitis in patients treated with plastic stents and patients treated with metal stents reporting a much higher incidence in the group treated with plastic stents, with a similar incidence of biliary leakage [6], but over time it is established As the most feared complication when using coated metal stents, the migration of the puncture site stent leaves an important defect open [6].

Finally, the USE-CD procedure is established using luminal apposing metal stent (HOT AXIOS) [3], that was initially developed for cases of drainage of pancreatic pseudocyst until being established in a biliary drainage procedure, particularly characterized by having short Length and weight form with wide eyelashes that allows anchoring through non-adherent structures, which infers the antimigratory property and the lower risk of biliary leakage [6,10,11], another important and very novel property is the inclusion of electrocautery and release of the stent that allows the accommodation of this in a single step thus reducing the replacement of instruments and therefore the number of complications [10,11].

The first multicenter study reporting the experience with cases of USE-CD with antimigratory stent (HOT AXIOS) is developed by Tsuchiya and collaborators in which they present a technical and clinical success of 100 and 95% respectively, but they had 5 patients from the total sample of 19 who developed stent occlusion in the next 184 days and conclude in advance the advent of more USE-CD [6].

As reviewed in the literature, the performance of endoscopic ultrasound-guided choledocoduodenostomy has an approximate percentage of adverse events of 16%, mainly consisting of infection, pneumoperitoneum, biliary leakage, bleeding, abdominal pain, perforation and stent migration, the most frequently reported being the Pneumoperitoneum with conservative management and good evolution of the patient [4,7], on the other hand the use of Doppler implies a very convenient weapon for the endoscopist when discarding the presence of vascular structure and avoiding complication [5], there are still reports of cases such as Mangas-Sanjuan and collaborators in which they present a case of accidental puncture of the portal vein at the time of performing a USE-CD, a complication that they managed to resolve by the same route obtaining control of bleeding and finally with clinical and technique success on Bile drainage [5,7].

To conclude, in our case we present the use of EUS-CD with LAMS (HOT AXIOS) in a patient with unresectable pancreatic cancer led to a failed ERCP due to the involvement of the Vater papilla, joining the report in the literature for high rates of technical and clinical success as discussed before.

Considering that EUS-BD is a safe procedure, with a high rate of technical and clinical success, low risk of complications, providing a better quality of life to the patient, should be performed by endoscopic gastroenterologists expert in EUS and in the use of LAMS [10,11]. It should be considered as the first choice on the PTCBD for biliary drainage when the ERCP fails especially in cases of biliary obstruction of malignant etiology.

## Declaration of Competing Interest

NA.

## Funding

NA.

## Ethical approval

Written informed consent was obtained from the patient for publication of this case report and accompanying images. A copy of the written consent is available for review by the Editor-in-Chief of this journal on request.

## Consent

Yes.

## Author contribution

Renzo Pinto-Carta: study concept or design, writing the paper, develop the procedure.

Jaime Solano: writing the paper, develop the procedure.

Felipe Cabrera: study concept or design.

Alvaro Sanchez: data collection, writing the paper.

Luisa Moreno: data collection: writing the paper.

Mauricio Pedraza: writing the paper.

## Registration of research studies

NA.

## Guarantor

Dr. Renzo Pinto-Carta. Gastroenterólogo intervencionista endosonografista.

Fundación Santa Fe De Bogotá. Bogotá Colombia.

E-mail: renso.pinto@fsfb.org.co.
